# How Portuguese Health Entities Used Social Media to Face the Public Health Emergency during COVID-19 Disease

**DOI:** 10.3390/ijerph191911942

**Published:** 2022-09-21

**Authors:** Daniela Azevedo, Ana Isabel Plácido, Maria Teresa Herdeiro, Fátima Roque, Vítor Roque

**Affiliations:** 1 Research Unit for Inland Development, Polytechnic of Guarda (UDI-IPG), 6300-559 Guarda, Portugal; 2 Institute of Biomedicine (iBiMED), Department of Medical Sciences, University of Aveiro, 3810-193 Aveiro, Portugal; 3 Health Sciences Research Centre, University of Beira Interior (CICS-UBI), 6200-506 Covilhã, Portugal

**Keywords:** communication, COVID-19 disease, health entities, interaction, public health emergency, social media

## Abstract

Background: During the COVID-19 pandemic, social media became an important and easily accessible source of information to keep the population informed. In this study, we explored how Portuguese health entities used social media during the public health emergency caused by COVID-19 disease. Methods: A retrospective study on Portuguese public health entities’ communication with the public using social media platforms was performed. Data were retrieved from Facebook, Instagram, and Twitter. All retrieved posts were analyzed, and the engagement of the public was calculated. A thematic analysis of all COVID-19-related posts was conducted. Results: The analysis of each social media platform revealed that during the COVID-19 pandemic, health entities reinforced their presence on social media platforms. The most published posts were related to “epidemiological context” and “encouragement to take actions” to avoid the spread of COVID-19. High engagement frames were not associated with the most frequently published posts. Conclusions: Although health entities have reinforced their presence on social media platforms, they do not take full advantage of these platforms to improve health literacy.

## 1. Introduction

Effective communication in health is the key to improving health literacy and promoting the health of the population. In the era of digital communication and digital health, the use of social media platforms can be an effective and quick approach to disseminating information related to public health [1]. Social media platforms are considered powerful health communication tools due to their capacity to portray the mainstream sources of information about health and wellness, and due to the ease of creating widespread public engagement [1]. Worldwide health entities have made efforts to use social media platforms to enhance communication [1]. Moreover, the engagement of the public through social media and two-way communication creates increased trust and positive feelings towards entities/institutions, and potentially reduces misinformation [2]. There is some evidence that public health agencies and health entities, in general, are using social media, but not exploiting their full potential for interaction [2].

At the beginning of 2020, coronavirus disease (COVID-19) became a severe public health problem worldwide, and governments and healthcare entities faced multiple challenges [3]. Governments needed to immediately plan and implement communication strategies that explain the measures that have been adopted and allow the management of public health campaigns [4]. This included finding a strategy to spread timely, accessible, and accurate information to all citizens without compromising public health.

In situations of crisis management, such as the COVID-19 pandemic, social media is a strategic and effective element that enables the quick and efficient dissemination of vital messages to the public about the reality of the situation, the consequences that derive from it, the personal and collective implications, and the behaviors, attitudes, and measures that must be taken [4,5]. In this scenario, social media platforms such as Facebook, Instagram, and Twitter were reported as the main ways of accessing news and receiving information related to health. Thus, utilizing these platforms has emerged as one effective method to communicate with the population, using frames related to public and personal safety to motivate the public to take part in preventative actions [6,7].

Social media gained importance during the lockdown when the population felt the urge to find information to tranquilize uncertain feelings about their future [3,8]. The relevance of studying how governments and health entities communicate in the context of a public health crisis—the COVID-19 pandemic—is linked to their responsibility to act swiftly to communicate crisis information to the public effectively and efficiently [9]. Recent studies suggested that during the COVID-19 pandemic, although governmental institutions of China, Spain, and the USA have made efforts to use social media platforms to communicate with the public, they failed in the promotion of public–government collaboration and engagement because the majority of the messages posted by governmental institutions were related to administrative or service information [4,9,10]. Studies suggested that on social media platforms, frames related to the impact of COVID-19 at a community or individual level have high public engagement [7,11,12].

In Portugal, the first case of COVID-19 was reported on 2 March 2020, and on 18 March 2020, the government declared the lockdown, and the Portuguese population used social media platforms to stay connected with the world.

Considering that more than 80% of the Portuguese population uses social media platforms and that Facebook, Instagram, and Twitter are among the ten most-used platforms by Portuguese internet users [13], we aimed to explore how Portuguese health entities used social media to communicate with citizens during the public health emergency caused by COVID-19 disease.

## 2. Materials and Methods

### 2.1. Study Design and Data Source

The Portuguese National Healthcare Service is public, free, and accessible to all Portuguese residents. Although considered one of the best healthcare services in the world [14], previous studies of our group found that in primary care services, the lack of communication is a gap that frequently compromises health outcomes [15,16]. For this reason, and considering that during the COVID-19 pandemic, the traditional channels of communication were restricted, and social media was considered the most effective and fastest channel of communication, we conducted a retrospective study on public health entities’ communication with the public using social media platforms.

Data on the presence, use, and engagement of social media users on Facebook, Instagram, and Twitter were retrieved between 1 January 2020 and 31 December 2020 from the official social media platforms of the selected health entities. The selection of health entities included in the study and their websites were obtained from the list provided by the national health service (SNS—*Serviço Nacional de Saúde*) website [17]. SNS is the Portuguese national authority on health that is responsible for ensuring the right to health and protection for all citizens [18]. SNS directly administers the directorate-general of health (DGS—*Direção-Geral da Saúde*) and indirectly administers the five regional health administration (ARS—*Administração Regional da Saúde*) entities [17]. The DGS regulates health promotion and disease prevention activities, and also defines the technical conditions for the adequate provision of healthcare and plans the national policy for quality in the health system [19]. The ARS entities guarantee that the population of a region has access to adequate healthcare services [20,21,22,23,24]. Although autonomous regions of Madeira and Azores are ruled by principles of the Portuguese national constitution, they have autonomy in the implementation of health policies. The regional directorate for the health of these islands is responsible for ensuring the execution of the regional plan of health [25]. The social media platforms of the autonomous regions’ health entities were retrieved from the official webpage of the two Portuguese regional executive governments [26,27].

**Figure 1:**
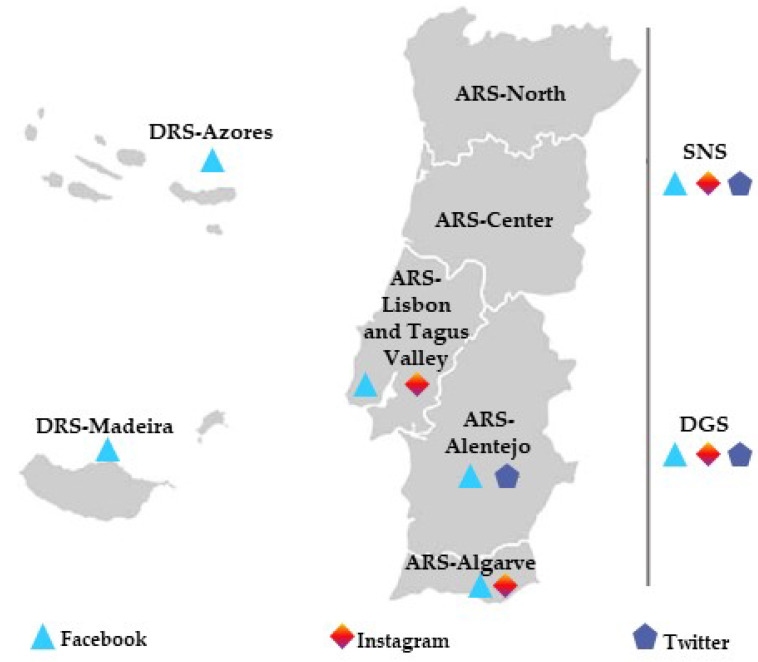
Presence of Portuguese health entities on social media.

Additionally, it was required for each Portuguese health entity (SNS, DGS, regional health administration of Alentejo (ARS-Alentejo), regional health administration of Algarve (ARS-Algarve), regional health administration of Center (ARS-Center), regional health administration of Lisbon and Tagus Valley (ARS-Lisbon and Tagus Valley), regional health administration of North (ARS-North), regional directorate for health of Azores (DRS-Azores), and regional directorate for health of Autonomous Region of Madeira (DRS-Madeira)) to confirm its official social media web page, if it exists (Figure 1). During the study period, two of the nine selected health entities (ARS-North and ARS-Center) did not have an official account on the analyzed platforms and were excluded from the analysis.

### 2.2. Data Collection

The extracted data included all posts published between 1 January 2020 and 31 December 2020 on each social media page/profile of the seven Portuguese health entities selected (SNS, DGS, ARS-Alentejo, ARS-Algarve, ARS-Lisbon and Tagus Valley, DRS-Azores, and DRS-Madeira).

Data were extracted between January and February 2022 by two independent researchers (D.A. and A.I.P.).

A Microsoft Excel dataset was constructed using the data extracted from all the official pages. Considering the possibility of a dependent operator error and to guarantee trustworthiness, the acquired data were revised one month after the last collection through a comparison of the data reported in the dataset with the screenshots taken at the time of collection.

The extracted data (Table 1) included post types (classified into four categories: image, video, publishing link, and text) and reactions (observed in three parameters: the number of likes, comments, and shares).

The engagement of the public with the social media platforms of health entities was assessed through the analysis of the user interaction, and for Facebook, it was calculated with the following formula [28]:

$$Facebook\;engagement=\;\frac{\left(total\_likes+total\_comments+total\_shares\right)}{\left(number\_posts\right)}$$

Based on the above mathematic expression portrayed in the literature for Facebook, the following formulas were deduced to calculate the engagement of Instagram and Twitter:

$$\begin{array}{c} Instagram\;engagement=\;\frac{\left(total\_likes+total\_comments\right)}{\left(number\_posts\right)}\\ Twitter\;engagement=\;\frac{\left(total\_likes+total\_retweets+total\_replies\right)}{\left(number\_tweets\right)} \end{array}$$

### 2.3. Thematic Analysis

To explore the main themes communicated by health entities on social media platforms and to obtain useful and credible frameworks of communication, a thematic analysis was performed following the methodology described by Braun and Clarke, which defines thematic analysis as “a method for identifying, analyzing and reporting patterns (themes) within data”. “It minimally organizes and describes data set in (rich) detail” [29]. Briefly, in a first step, to allow a better understanding of the content of all posts, two researchers read, multiple times, all COVID-19-related posts and created the initial “coding concepts” (Table 2). Then, the codes were grouped into ten different themes. After that, the themes were revised and data were interpreted and discussed by all members of the research team. After an accurate analysis of the emerged themes and considering previously published studies [6,7,30], the research team followed the strategy used by Kandzer et al. [6] and created a new theme, “community and protecting yourself” (Table 2). This new theme encompasses all the posts that contain a message directed simultaneously at the community and individuals.

## 3. Results

### 3.1. Health Entities and Social Media Accounts

It was observed that of the seven health entities (SNS, DGS, ARS-Alentejo, ARS-Algarve, ARS-Lisbon and Tagus Valley, DRS-Azores, and DRS-Madeira) included in this study, five of them used at least two social media platforms to communicate (Supplementary Materials Table S1).

The social media platform most frequently used is Facebook, used by all health entities, followed by Instagram, used by four health entities. Only three health entities used Twitter to communicate. The analysis of the different social media uniform resource locators (URLs) used by each ARS revealed a pattern with the official website and consistency between the different platforms (Supplementary Materials Table S1). No consistency was observed for the website and social media URL used by the Autonomous Region of Madeira.

The analysis of the total posts published by all entities on the three social media platforms (Facebook, Instagram, and Twitter) revealed that SNS and DGS were the health entities with the highest median number of posts per month (131.8 and 58.0, respectively). In both health entities, a huge increase in the number of posts was observed in March, and this increase remained stable during the data collection period (Supplementary Materials Table S2).

The number of posts on social media platforms of the ARS ranged from 8.3 to 24.5, ARS-Algarve being the least active on the social media platforms. On average, ARS-Lisbon and Tagus Valley had 21.8 posts per month, with a similar trend of monthly publications with SNS and DGS. In the first four months of the year, the pattern of posts of ARS-Alentejo was also similar to SNS and DGS, with a tendency to increase. In the remaining months, the presence of this health entity on social media platforms decreased, reaching a minimum number of posts per month (15.5) in December. The median number of posts per month observed on the social media platforms of DRS-Madeira and DRS-Azores was 38.5 and 27.0, respectively. In DRS-Azores, the number of posts increased 10-fold between January and February and started to decrease in April. In August and September, this health entity did not register any posts on social media platforms (Supplementary Materials Table S2).

All posts of the five health entities in the study on three social media platforms (Facebook, Instagram, and Twitter) were grouped into COVID-19-related or non-COVID-19-related posts. The monthly analysis (after March) revealed that the majority of the SNS and DGS posts were COVID-19-related (Supplementary Materials Table S3). 

### 3.2. Thematic Analysis

Major themes emerged from our detailed thematic analysis of the COVID-19-related posts, described in Table 2.

**Figure 2:**
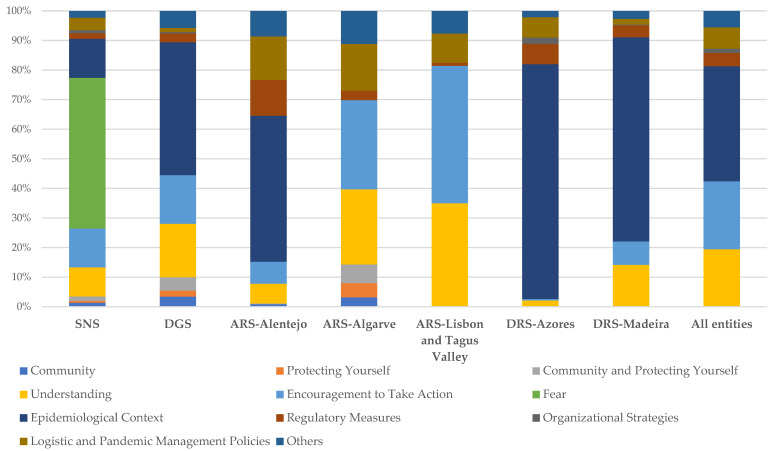
Emerged themes from the thematic analysis of health entities’ posts on Facebook, Instagram, and Twitter.

For the set of social media platforms of the entities under study, “epidemiological context”, “encouragement to take action”, and “understanding” were the themes most reported, with post percentages of 34.6%, 20.4%, and 17.3%, respectively. On the other hand, “organizational strategies”, “protecting yourself”, and “community” were the themes with the lowest number of posts, with post percentages of only 1.3%, 1.6%, and 2.7%, respectively (Supplementary Materials Table S4). Figure 2 shows the weighting of each theme in the total number of publications per entity.

#### 3.2.1. National Health Service

The majority (59.9%) of the Facebook posts of the Portuguese national health service (SNS) were assigned to the theme “epidemiological context” (Table 3). The themes “logistic and pandemic management policies” and “encouragement to take action” represented 8.2% and 6.7% of the Facebook posts, respectively (Table 3). The remaining themes were minor Facebook posts, each representing less than 5% of the published posts. Among the emerged themes, “community and protecting yourself”, “community”, and “understanding” obtained the highest interaction values (569.2, 552.3, and 545.7, respectively) (Table 3). “Logistic and pandemic management policies” and “organizational strategies” were the themes that generated lower interaction from the public (Table 3).

Links were the preferred type of post used by SNS in posts related to the themes “encouragement to take action”, “regulatory measures”, “organizational strategies”, and “logistic and pandemic management policies” (Supplementary Materials Table S5). The remaining themes were primarily posted as images (Supplementary Materials Table S5). Although the majority of the posts on this platform were images, videos generated the highest interaction (Supplementary Materials Table S5). Most themes posted on SNS-Instagram were related to “encouragement to take action” (39.7%), “understanding” (31.7%), “fear” (5.9%), and “community and protecting yourself” (5.6%) (Table 3). The themes “epidemiological context” and “organizational strategies” were the least published (Table 3). “Regulatory measures” and “logistic and pandemic management policies” generated the highest interaction values (1517.3 and 1021.1, respectively) (Table 3). Regarding SNS-Twitter publications, 26.9%, 24.1%, and 17.9% were related to “encouragement to take action”, “epidemiological context”, and “understanding”, respectively (Table 3). Themes related to individual and/or community protection were minor on the social media webpage, representing less than 6% of all publications (Table 3). The themes “fear” and “community” obtained the highest interaction values (28.2 and 24.1, respectively) (Table 3). On both Twitter and Instagram, the majority of the themes were published as images (Supplementary Materials Table S5).

#### 3.2.2. Directorate-General of Health

During the analyzed period, more than 75% of DGS Facebook posts were associated with the “epidemiological context” (Table 3). Although being one of the least published themes (0.8%), “logistic and pandemic management policies” had the highest number of public interactions (27,442) (Table 3).

On Instagram, DGS focused its publications on the promotion of the “understanding” of COVID-19 and on the “encouragement to take action” of the target public (Table 3). Although only 0.4% of Instagram content was related to “epidemiological context”, this theme had the highest number of interactions on this social media platform (Table 3).

On Twitter, “fear”-related content represented more than 50.0% of all published posts by DGS, followed by “understanding” (15.6%) and by content encouraging the public to take measures to protect the community and oneself (Table 3). On this social media platform, the theme “community” represented only 2.7% of the published posts and achieved the highest interaction value (140.3) (Table 3). Image content was privileged by DGS on all social media platforms used (Supplementary Materials Table S5).

#### 3.2.3. Regional Health Administrations

##### ARS-Alentejo

More than 69.0% of the posts published by ARS-Alentejo on Facebook were classified as “epidemiological context” (Table 3). This ARS did not publish any content related to the themes “protecting yourself”, “community and protecting yourself”, “fear”, and “organizational strategies”. The highest value of public interaction was observed for the “encouragement to take action” theme (43.1) (Table 3). Regarding the type of publication, although the majority of the posts published by this ARS were images (79.3%), for the themes “community”, “understanding”, “encouragement to take action”, “regulatory measures”, “logistic and pandemic management policies”, and “others”, the dominant type of post was links (Supplementary Materials Table S5). On Twitter, this ARS focused their published content on “logistic and pandemic management policies” (31.1%) and “regulatory measures” (28.3%) (Table 3). On this social media platform, ARS-Alentejo published exclusively text content (Supplementary Materials Table S5).

##### ARS-Algarve

During the study period, the main posts published on Facebook were related to the themes “encouragement to take action” (33.3%), “understanding” (22.8%), and “logistic and pandemic management policies” (14.7%) (Table 3). No posts related to “epidemiological context” and “organizational strategies” were published by this ARS. The posts that generated the highest public interaction were “logistic and pandemic managing policies” (67.9%) (Table 3). On Facebook, the type of publication varied under the published theme; for example, “community” and “epidemiological context” were published exclusively as images (Supplementary Materials Table S5), and “regulatory measures”, “logistic and pandemic management policies”, and “others” were most frequently posted as links (Supplementary Materials Table S5).

On Instagram, the themes “community and protecting yourself” and “logistic and pandemic management policies” were the most posted (Table 3). On this social media platform, themes such as “protecting yourself”, “encouragement to take action”, and “epidemiological context” were not mentioned (Table 3). Regarding the type of publication, ARS-Algarve privileged image content to communicate on this platform (Supplementary Materials Table S5).

##### ARS-Lisbon and Tagus Valley

The thematic analysis of the Facebook posts of ARS-Lisbon and Tagus Valley demonstrated that 33.6% of their posts were associated with the theme “encouragement to take action” (Table 3). “Understanding” accounted for 19.6% of the posts (Table 3). On Facebook, “logistic and pandemic management policies” was the theme that generated the highest interaction (1955.9) (Table 3). On Instagram, a similar pattern of published posts and interaction was observed for this ARS (Table 3). The majority of the posts published by this ARS on social media were images (Supplementary Materials Table S5).

#### 3.2.4. Regional Directorate for Health

##### DRS-Azores

It was observed that more than 79% of the published posts of DRS-Azores on Facebook were related to the “epidemiological context” theme (Table 3). Themes related to individual or community protection were not published by this ARS (Table 3). Although only 2.1% of the content was related to “understanding”, this theme generated the highest value of public interaction (1314.3) (Table 3). DRS-Azores primarily used image content to communicate themes such as “understanding”, “epidemiological context”, and “logistic and pandemic management policies” (Supplementary Materials Table S5). The theme “encouragement to take actions” was exclusively published in video publications (Supplementary Materials Table S5).

##### DRS-Madeira

On Facebook, the main themes published by this DRS were “epidemiological context” (61.6%), followed by “understanding” (12.7%) (Table 3). The highest number of interactions was observed for the theme “fear” (Table 3). The majority of the publications shared by DRS-Madeira were videos (Supplementary Materials Table S5).

## 4. Discussion

This study showed that the COVID-19 pandemic led Portuguese health entities to reinforce their communication on social media platforms. Because the most published themes were not the themes that obtained the highest values of public engagement, health entities should re-think their way of communication to ensure efficient and effective communication with the public.

The public health risk associated with the COVID-19 pandemic led health authorities to find new strategies to communicate with the population. Studies published before the COVID-19 pandemic highlighted the advantage of digital platforms, and social media platforms have emerged as the natural solution [31]. However, the pandemic has also highlighted the need to investigate measures to mitigate the negative effects of these resources [32]. Among the negative effects of social media platforms that should be mitigated by national healthcare services, we highlight the high volume of information in a short period posted on social media platforms that can overwhelm the public trying to discern facts from false information [33,34]. The usability of social media in public health promotion was observed before the COVID-19 pandemic in several public health initiatives, such as smoking cessation and cancer prevention campaigns, and also epidemiological monitoring during the H7N9 avian influenza virus outbreak [35,36,37]. In this study, it was observed that the two regional health entities did not use any social media platform (ARS-North and ARS-Center), and the remaining regional health entities used only two of the three platforms, suggesting that Portuguese health entities did not take full advantage of social media platforms. This behavior may have complicated the communication between the public and health authorities and, consequently, may have negatively influenced the attitudes and beliefs of the population towards reducing the COVID-19 spread [38]. Because the Portuguese population trusts health entities and the information disclosed by health entities [39], we believed that health entities’ social media webpages have the potential to be public health literacy tools.

Although before the first reported COVID-19 case, the presence of health entities on social media was subtle, with regional health entities publishing less than one post per month, after the first reported COVID-19 case in Portugal, health entities improved efforts to communicate through the reinforcement of their presence on social media platforms, increasing the number of posts.

The thematic analysis suggests that Portuguese health entities followed the guidelines suggested in the “COVID-19 toolkits” [40] of the Centers for Disease Control and Prevention (CDC), using simple, direct, and effective messages to encourage the community and individuals to take action. Several similarities were found between the strategies observed in this study and the CDC communication guidelines [6].

Health entities adapted the frames to the social media platform used. On Facebook, the national authorities (SNS and DGS) and the regional authorities of the autonomous regions of Madeira and Azores devoted more than 50% of their posts to reporting the epidemiological updates of the COVID-19 pandemic; ARS-Algarve adopted an identical strategy. The remaining ARSs focused their Facebook posts on the themes “understanding” and “encouragement to take action”. Epidemiological updates were also the most frequently published posts of Singapore’s Ministry of Health [41] and the Chinese government [10]. Sentences related to the theme “encouragement to take action” were also one of the most common frames that emerged from the thematic analysis of the Facebook page of the U.S. Centers for Disease Control and Prevention (CDC) [6]. The thematic analysis of the Instagram account of the selected health entities revealed that on this social media platform, “understanding” and “encouragement to take action” were the most frequently published frames. On Twitter, different frames were published by the different ARSs. The “epidemiological context” was the most tweeted frame by SNS, while fear-associated messages represented more than 50% of the published posts of DGS. ARS-Alentejo devoted the majority of its tweets to “logistic and pandemic management policies” and “regulatory measures”. The frames related to “community” and “protecting yourself” were the least posted by the national health entities SNS and DGS; however, these frames generated the highest number of interactions. These data suggest that despite health entities having increased the volume of posts, the frames lack adjustment to the target audience. At the regional level, frames related to “logistic and pandemic management policies” generated the highest public interaction. As social media interactions were considered a promising means of advancing person-to-person engagement with science [30], high interaction can be interpreted as an indicator of the engagement and interest of the public. In this case, high interaction reveals a reaction of the public to the increase in resources provided at a local level to help health professionals manage the COVID-19 pandemic.

On Twitter, Jordan et al. [7] compared the effectiveness of COVID-19 frames focused on an “individual level”, “community level”, and both “community and individual level”, and observed that frames focused on statements related to the impact of COVID-19 at a community level were more effective than individual-level COVID-19 messages [7]. On Facebook, the data obtained by Banker et al. suggest that frames focused on protecting oneself were the most effective [11]. Although a previous study suggested that fear-associated frames must be avoided, these statements were frequently used by the selected entities [42]. A recent study suggested that during a public health crisis, the public is more interested in the textual content of a frame than whether it contains a video or a picture, and the majority of the posts on the Portuguese health entities’ social accounts were images [10]. Another study reported that text posts were disliked by Twitter users, who preferred video posts, but favored by Facebook users [38].

This study provides relevant information to health stakeholders to develop new strategies to improve their communication on social media platforms, but it has some limitations. The study should cover the entire pandemic period to allow an understanding of how health entities’ communication processes changed during the pandemic period and how the public reacted. Because the retrieved data do not consider the number of followers and the demographic characteristics of the engaged public, we cannot infer the characteristics of the population that interacts with the social media health entities’ webpages. We also cannot verify if the communication of health entities on social media platforms reached the majority of the population or not.

## 5. Conclusions

This study suggests that during the first year of the COVID-19 pandemic, Portuguese authorities increased their presence on social media platforms, but the content of the publications is not in line with the engagement of the public, suggesting that health entities do not take full advantage of social media platforms to spread information regarding the behaviors of the population to avoid the spread of COVID-19.

Considering that communication between all players of health services is fundamental to ensuring adequate healthcare services, the data provided by this study are useful to help Portuguese health stakeholders develop new strategies to improve the communication between public and health entities.

The thematic analysis can be useful to establish foundational frameworks for research on social media communication during public health emergencies.

## Figures and Tables

**Table 1:** Post types by social media.

		Facebook	Instagram	Twitter
Post types	Image	X	X	X
	Video	X	X	X
	Publishing link	X	-	X
	Text	X	-	X
Reactions	Likes	X	X	X
	Comments	X	X	X
	Shares	X	-	X

X: Types of posts/reactions present in social media.

**Table 2:** Major themes from health entities’ posts on social media.

Major Themes	Description	Coding Concepts	Quotes
Community	Incentives for the public to protect others by adopting preventative actions to help reduce the spread of COVID-19.	Staying home, social distancing, hand hygiene, respiratory etiquette, and aeration of spaces.	*“when you cough or sneeze, you release droplets, secretions, or aerosols that can be inhaled by other people or deposited on objects and surfaces around you”*
Protecting Yourself	Incentives for actions to protect oneself from contracting COVID-19.	Wearing a face mask, physical distancing, hand washing and disinfection, no object or food sharing, and avoiding touching surfaces.	*“some simple gestures make a difference in protecting against COVID-19, it is very important to disinfect your phone”*
Community and Protecting Yourself	Incentives for the public to protect others by adopting preventative actions to help reduce the spread of COVID-19 and also actions to protect oneself from contracting COVID-19.	Protecting yourself and those around you, being a public health agent, preventing the spread of the virus, protecting yourself and others, and reducing the risk of disease exposure and transmission.	*“the thought must be: when I comply with the recommended measures, I am protecting not only myself but also the people who live with me, my family, my friends”.*
Understanding	Statements of a level of understanding about COVID-19 disease and the best practices to follow to keep safe.	Understanding, meet, know that, know more, remember, recommendations, and instructions.	*“coronaviruses are a family of viruses known to cause illness in humans and the infection can be similar to the common flu or present as a more serious illness such as pneumonia”*
Encouragement to Take Action	Public motivation to take action to stay safe from COVID-19.	Appeals and indications of public figures and institutions to follow the example of, awareness campaigns to stop the pandemic, and videos that teach preventative measures and the correct ways to exercise them.	*“only with everyone’s commitment is it possible to stop the transmission of this virus, thank you for your contribution”*
Fear	Statements related to the risk and severity of COVID-19.	The virus is still active, the virus still has not disappeared, you do not see it, but it continues to circulate, the virus does not take a vacation, and with COVID-19, all care is little.	*“sharing objects increases the risk of transmitting the virus”*
Epidemiological Context	Data about the number of infections and information on the spread of COVID-19.	COVID-19 press conference, today’s status report, epidemiological situation assessment in Portugal, and Public Health Authorities’ communiqués.	*“COVID-19 press conference” and “today’s status report is now available”*
Regulatory Measures	Data and information about government rules and measures related to COVID-19 disease and its spread.	Extraordinary measures, restrictions, sanitation fence, support measures package, vaccination priorities, standards and technical guidelines, state of emergency, calamity, and contingency.	*“the European Commission authorized the first COVID-19 vaccine for use in the European Union”*
Organizational Strategies	Information about actions, activities, and partnerships of the government.	Meetings, visits, and creation of commissions and task forces.	*“government creates task-force to prepare the vaccination plan”*
Logistic and Pandemic Management Policies	Strategies and improvements applied to services and infrastructures and approaches to the field of pandemic management.	Reinforce response capacity, expansion of laboratory capacity, update contingency plans in health units, and human resources hiring.	*“hospital pharmacy: medicines delivered to pharmacies and at home to avoid trips to hospitals”*
Others	Variated information that cannot be included in any theme aforementioned.	National programs, manuals disclosure, awards, distinctions, etc.	*“the Directorate-General for Health (DGS) and the Shared Services of the Ministry of Health (SPMS) were distinguished by the International Hospital Federation-IHF, in the context of combating the pandemic, with the project ‘Self-Report&TraceCOVID-19′”*

**Table 3:** Proportion and interaction of major themes from the health entities’ posts on Facebook, Instagram, and Twitter.

		SNS			DGS			ARS-Alentejo		ARS-Algarve		ARS-Lisbon and Tagus Valley		DRS-Azores	DRS-Madeira
		Facebook	Instagram	Twitter	Facebook	Instagram	Twitter	Facebook	Twitter	Facebook	Instagram	Facebook	Instagram	Facebook	Facebook
Community	%	1.5%	4.0%	2.5%	2.4%	4.8%	2.7%	1.2%	-	1.7%	9.1%	4.7%	5.6%	-	1.8%
	Interaction	552.3	649.2	24.1	7446.0	214.7	140.3	6.0	-	15.0	18.0	48.8	5.1	-	46.0
Protecting Yourself	%	1.7%	1.2%	0.9%	0.2%	4.6%	1.1%	-	-	5.3%	-	5.6%	5.6%	-	3.2%
	Interaction	364.2	617.9	19.7	17271.0	151.2	68.0	-		14.3	-	50.2	3.4	-	141.3
Community and Protecting Yourself	%	1.8%	5.6%	2.3%	1.3%	8.1%	12.6%	-	0.9%	3.5%	18.2%	4.7%	4.0%	-	3.5%
	Interaction	569.2	661.3	22.4	3520.2	141.0	63.1	-	0.0	22.5	26.5	86.0	7.0	-	42.1
Understanding	%	7.3%	31.7%	17.9%	5.7%	33.8%	15.6%	4.7%	11.3%	22.8%	27.3%	19.6%	34.4%	2.1%	12.7%
	Interaction	545.7	616.0	24.2	6360.4	149.8	87.3	11.1	0.0	26.2	11.3	56.2	5.3	1314.3	146.4
Encouragement to Take Action	%	6.7%	39.7%	26.9%	5.7%	25.6%	3.9%	7.0%	8.5%	33.3%	-	33.6%	39.2%	0.5%	7.1%
	Interaction	421.0	768.4	24.1	7829.6	182.9	79.7	43.1	0.0	46.4	-	55.3	5.9	261.0	63.6
Fear	%	4.3%	5.9%	2.4%	1.7%	7.5%	51.2%	-	0.9%	5.3%	18.2%	5.6%	6.4%	-	2.1%
	Interaction	267.0	4.0	28.2	5628.3	149.9	58.7	-	0.0	18.7	16.0	43.7	5.8	-	234.0
Epidemiological Context	%	59.9%	0.8%	24.1%	77.3%	0.4%	4.1%	69.1%	0.9%	-	-	-	-	79.4%	61.6%
	Interaction	298.3	592.2	15.1	3507.7	230.0	69.8	0.4	0.0	-	-	-	-	217.0	14.0
Regulatory Measures	%	3.0%	2.0%	4.9%	1.3%	2.9%	0.9%	5.5%	28.3%	3.5%	-	0.9%	0.8%	6.9%	3.5%
	Interaction	256.1	1517.3	16.7	4322.8	92.4	39.8	2.1	0.0	10.0	-	282.0	9.0	275.0	50.2
Organizational Strategies	%	2.7%	0.8%	2.5%	0.4%	-	1.9%	-	-	-	-	-	-	2.1%	0.3%
	Interaction	212.5	522.7	9.4	2287.5	-	62.4	-	-	-	-	-	-	71.8	12.0
Logistic and Pandemic Management Policies	%	8.2%	4.2%	10.4%	0.7%	1.3%	2.5%	7.8%	31.1%	12.3%	27.3%	15.9%	0.8%	6.9%	1.8%
	Interaction	136.3	1021.1	12.6	27442.0	82.5	45.9	16.3	0.1	67.9	38.7	1955.9	25.0	533.7	145.8
Others	%	2.9%	4.2%	5.2%	3.3%	11.0%	3.7%	4.7%	17.9%	12.3%	-	9.4%	3.2%	2.1%	2.4%
	Interaction	439.0	651.7	16.0	3162.4	90.8	40.4	16.3	0.0	21.0	-	52.7	6.3	406.3	43.4
Total Themes	%	100.0%	100.0%	100.0%	100.0%	100.0%	100.0%	100.0%	100.0%	100.0%	100.0%	100.0%	100.0%	100.0%	100.0%
	Interaction	320.3	708.1	19.6	4226.1	152.0	72.2	6.0	0.0	35.5	23.0	359.5	5.7	267.2	50.1

ARS-Alentejo—Regional Health Administration of Alentejo; ARS-Algarve—Regional Health Administration of Algarve; ARS-Lisbon and Tagus Valley—Regional Health Administration of Lisbon and Tagus Valley; DGS—Directorate-General of Health; DRS-Azores—Regional Directorate for Health of the Azores; DRS-Madeira—Regional Directorate for Health of the Autonomous Region of Madeira; SNS—National Health Service.

## Data Availability

Data were collected from public social media platforms (www.facebook.com/sns.gov.pt, www.instagram.com/sns_pt, www.twitter.com/SNS_Portugal, www.facebook.com/direcaogeralsaude, www.instagram.com/direcao_geral_saude, www.twitter.com/DGSaude, www.facebook.com/arsalentejo, www.twitter.com/arsalentejo, www.facebook.com/ARSAlgarveIP, www.instagram.com/ars_algarve, www.facebook.com/ARSLVT, www.instagram.com/ars_lvt, www.facebook.com/DirecaoSaudeAcores and www.facebook.com/profile.php?id=100069232239019) (accessed on 28 December 2021).

## Supplementary Materials

The following supporting information can be downloaded at: https://www.mdpi.com/article/10.3390/ijerph191911942/s1, Table S1: Health entities’ presence on social media platforms and analysis of respective uniform resource locators (URLs); Table S2: Health entities’ presence on social media platforms: number of posts per month and median number of posts; Table S3: Percentage of COVID-19-related and non-COVID-19-related posts of health entities per month on social media platforms/accounts, Table S4: Major emerged themes from the health entities’ posts on Facebook, Instagram, and Twitter, Table S5: Interaction with major emerged themes from the health entities’ posts on Facebook, Instagram, and Twitter by type of post.
