# Supplementary material for: How Portuguese Health Entities Used Social Media to Face the Public Health Emergency during COVID-19 Disease
Source: Int J Environ Res Public Health. 2022 Sep 21;19(19):11942. doi: 10.3390/ijerph191911942 (PMC9564587; doi:10.3390/ijerph191911942)
Supplement: Supplementary file 1 [file ijerph-19-11942-s001.zip › ijerph-1876737-supplementary.pdf]

Table S1. Health Entities' presence on social media platforms and analysis of respective Uniform Resource Locator (URL).

Health Entities' webpages	Social Media Accounts		
	**Facebook**	**Instagram**	**Twitter**
**SNS** www.sns.gov.pt	www.facebook.com/sns.gov.pt	www.instagram.com/sns_pt	www.twitter.com/SNS_Portugal
**DGS** www.dgs.pt	www.facebook.com/direcaogeralsaude	www.instagram.com/direcao_geral_saude	www.twitter.com/DGSaude
**ARS-Alentejo** www.arsalentejo.min-saude.pt	www.facebook.com/arsalentejo	-	www.twitter.com/arsalentejo
**ARS-Algarve** www.arsalgarve.min-saude.pt/	www.facebook.com/ARSAlgarveIP	www.instagram.com/ars_algarve	-
**ARS-Center** www.arscentro.min-saude.pt	-	-	-
**ARS-Lisbon and Tagus Valley** www.arslvt.min-saude.pt	www.facebook.com/ARSLVT	www.instagram.com/ars_lvt	-
**ARS-North** www.arsnorte.min-saude.pt	-	-	-
**DRS-Azores** www.portal.azores.gov.pt/pt/web/drs	www.facebook.com/DirecaoSaudeAcores	-	-
**DRS-Madeira** www.madeira.gov.pt/srs	www.facebook.com/profile.php?id=100069232239019	-	-

Table S2. Health Entities' presence on social media platforms: posts number per month and median posts number.

	**January**	**February**	**March**	**April**	**May**	**June**	**July**	**August**	**September**	**October**	**November**	**December**	**median**
**SNS**	75.7	67.0	117.0	141.0	153.3	141.3	147.7	132.7	150.0	122.0	130.0	131.0	131.8
**DGS**	1.7	3.7	50.3	76.3	76.3	62.7	71.3	56.7	53.0	56.0	72.3	59.3	61.3
**ARS-Alentejo**	16.0	13.5	23.5	52.5	39.0	34.0	29.5	18.5	33.5	26.5	23.5	15.5	24.5
**ARS-Algarve**	12.5	10.0	3.5	8.0	11.5	8.5	6.0	3.0	8.0	5.5	8.5	8.5	8.3
**ARS-Lisbon and Tagus Valley**	4.0	1.0	3.0	5.5	12.0	30.5	25.5	29.5	23.0	32.5	36.0	20.5	21.8
**DRS-Azores**	2.0	19.0	41.0	38.0	22.0	23.0	10.0	0.0	0.0	3.0	6.0	54.0	27.0
**DRS-Madeira**	5.0	9.0	24.0	68.0	65.0	45.0	52.0	28.0	49.0	46.0	50.0	28.0	38.5

ARS-Alentejo - Regional Health Administration of Alentejo; ARS-Algarve - Regional Health Administration of Algarve; ARS-Lisbon and Tagus Valley - Regional Health Administration of Lisbon and Tagus Valley; DGS - Directorate-General of Health; DRS-Azores - Regional Directorate for Health of the Azores; DRS-Madeira - Regional Directorate for Health of the Autonomous Region of Madeira; SNS - National Health Service.

Table S3. COVID-19-Related and no COVID-19-Related posts percentage of Health Entities per month on social media platforms/accounts.

		**January**	**February**	**March**	**April**	**May**	**June**	**July**	**August**	**September**	**October**	**November**	**December**	**2020**
**SNS**	COVID-19-Related	5.4%	22.2%	91.7%	90.4%	81.9%	65.9%	56.3%	63.7%	60.0%	64.4%	84.0%	89.4%	67.6%
	No COVID-19-Related	94.6%	77.8%	8.3%	9.6%	18.1%	34.1%	43.7%	36.3%	40.0%	35.6%	16.0%	10.6%	32.4%
**DGS**	COVID-19-Related	100.0%	50.0%	100.0%	83.1%	81.5%	86.8%	85.4%	97.4%	83.8%	67.8%	82.5%	88.3%	84.5%
	No COVID-19-Related	0.0%	50.0%	0.0%	16.9%	18.5%	13.2%	14.6%	2.6%	16.2%	32.2%	17.5%	11.7%	15.5
**ARS-Al**	COVID-19-Related	0.0%	25.0%	62.5%	77.1%	36.5%	78.6%	77.1%	79.2%	60.0%	60.0%	56.8%	71.0%	61.7%

	No COVID-19-Related	100.0%	75.0%	37.5%	22.9%	63.5%	21.4%	22.9%	20.8%	40.0%	40.0%	43.2%	29.0%	38.3%
**ARS-Algarve**	COVID-19-Related	5.9%	13.3%	85.7%	53.3%	52.2%	40.0%	41.7%	50.0%	18.18%	12.5%	26.67%	50.00%	34.8%
	No COVID-19-Related	94.1%	86.7%	14.3%	46.7%	47.8%	60.0%	58.3%	50.0%	81.82%	87.5%	73.33%	50.00%	65.2%
**ARS-Lisbon and Tagus Valley**	COVID-19-Related	37.5%	0.0%	50.0%	54.5%	12.5%	56.2%	48.2%	50.0%	35.0%	19.1%	57.6%	63.6%	42.3%
	No COVID-19-Related	62.5%	100.0%	50.0%	45.5%	87.5%	43.8%	51.8%	50.0%	65.0%	80.9%	42.4%	36.4%	57.7%
**DRS-Azores**	COVID-19-Related	100.0%	15.8%	90.2%	94.7%	86.4%	95.6%	100.0%	-	-	33.3%	100.0%	98.1%	86.7%
	No COVID-19-Related	0.0%	84.2%	9.8%	5.3%	13.6%	4.4%	0.0%	-	-	66.7%	0.0%	1.9%	13.3%
**DRS-Madeira**	COVID-19-Related	60.0%	77.8%	95.8%	91.2%	50.8%	88.9%	73.1%	64.3%	51.0%	56.5%	78.0%	89.3%	72.3%
	No COVID-19-Related	40.0%	22.2%	4.2%	8.8%	49.2%	11.1%	26.9%	35.7%	49.1%	43.5%	22.0%	10.7%	27.7%

ARS-Alentejo - Regional Health Administration of Alentejo; ARS-Algarve - Regional Health Administration of Algarve; ARS-Lisbon and Tagus Valley - Regional Health Administration of Lisbon and Tagus Valley; DGS - Directorate-General of Health; DRS-Azores - Regional Directorate for Health of the Azores; DRS-Madeira - Regional Directorate for Health of the Autonomous Region of Madeira; SNS - National Health Service.

Table S4. Emerged major themes percentage from the Health Entities' posts on Facebook, Instagram and Twitter.

	**SNS**	**DGS**	**ARS-Alentejo**	**ARS-Algarve**	**ARS-Lisbon and Tagus Valley**	**DRS-Azores**	**DRS-Madeira**	**All Entities**
**Community**	2.7%	3.2%	0.8%	3.0%	5.2%	-	1.8%	2.7%
**Protecting Yourself**	1.1%	1.9%	-	4.4%	5.6%	-	3.2%	1.6%
**Community and Protecting Yourself**	3.1%	4.4%	0.3%	5.9%	4.3%	-	3.5%	3.2%
**Understanding**	19.2%	17.2%	6.6%	23.5%	27.6%	2.1%	12.7%	17.3%
**Encouragement to Take Action**	25.8%	15.8%	7.5%	27.9%	36.6%	0.5%	7.1%	20.4%
**Fear**	3.8%	4.4%	0.3%	7.4%	6.0%	-	2.1%	3.6%
**Epidemiological Context**	25.9%	43.0%	49.2%	-	-	79.4%	61.6%	34.6%
**Regulatory Measures**	3.7%	2.9%	12.1%	2.9%	0.9%	6.9%	3.5%	4%
**Organizational Strategies**	2.1%	0.5%	-	-	-	2.1%	0.3%	1.3%
**Logistic and Pandemic Management Policies**	8.2%	1.3%	14.6%	14.7%	7.8%	6.9%	1.8%	6.4%
**Others**	4.4%	5.4%	8.6%	10.3%	6.0%	2.1%	2.4%	4.9%

ARS-Alentejo - Regional Health Administration of Alentejo; ARS-Algarve - Regional Health Administration of Algarve; ARS-Lisbon and Tagus Valley - Regional Health Administration of Lisbon and Tagus Valley; DGS - Directorate-General of Health; DRS-Azores - Regional Directorate for Health of the Azores; DRS-Madeira - Regional Directorate for Health of the Autonomous Region of Madeira; SNS - National Health Service.

Table S5. Emerged major themes percentage and interaction from the Health Entities' posts on Facebook, Instagram, and Twitter: by type of post.

			SNS			DGS			ARS-Alentejo		ARS-Algarve		ARS-Lisbon and Tagus Valley		DRS-Azores	DRS-Madeira
			Facebook	Instagram	Twitter	Facebook	Instagram	Twitter	Facebook	Twitter	Facebook	Instagram	Facebook	Instagram	Facebook	Facebook
Community	Image	%	90.0%	61.3%	66.7%	81.8%	91.3%	93.3%	-	-	-	100.0%	100.0%	100.0%	-	83.3%
		Interaction	567.4	778.6	24.3	7762.7	227.1	143.2	-	-	-	18.0	48.8	5.1	-	38.0
	Video	%	-	38.7%	25.0%	18.2%	8.7%	6.7%	-	-	100.0%	-	-	-	-	-
		Interaction	-	444.4	28.9	6021.0	85.0	100.0	-	-	15.0	-	-	-	-	-
	Publishing link	%	10.0%	-	8.3%	-	-	-	100.0%	-	-	-	-	-	-	16.7%
		Interaction	416.0	-	8.0	-	-	-	6.0	-	-	-	-	-	-	86.0
	Text	%	-	-	-	-	-	-	-	-	-	-	-	-	-	-
		Interaction	-	-	-	-	-	-	-	-	-	-	-	-	-	-
Protecting Yourself	Image	%	90.9%	100.0%	92.3%	100.0%	90.9%	83.3%	-	-	66.7%	-	100.0%	100.0%	-	100.0%
		Interaction	383.4	617.9	20.8	17271.0	159.3	62.8	-	-	10.0	-	50.2	3.4	-	141.3
	Video	%	-	-	-	-	9.1%	-	-	-	33.3%	-	-	-	-	-
		Interaction	-	-	-	-	70.5	-	-	-	23.0	-	-	-	-	-
	Publishing link	%	9.1%	-	7.7%	-	-	16.7%	-	-	-	-	-	-	-	-
		Interaction	172.0	-	6.0	-	-	94.0	-	-	-	-	-	-	-	-
	Text	%	-	-	-	-	-	-	-	-	-	-	-	-	-	-
		Interaction	-	-	-	-	-	-	-	-	-	-	-	-	-	-
Community and Protecting Yourself	Image	%	75.0%	93.2%	94.1%	83.3%	92.3%	95.2%	-	-	50.0%	50.0%	80.0%	100.0%	-	91.7%
		Interaction	435.7	603.4	20.7	4038.6	143.3	64.4	-	-	30.0	29.0	100.5	7.0	-	43.0
	Video	%	16.7%	6.8%	2.9%	-	7.7%	-	-	-	50.0%	50.0%	20.0%	-	-	-
		Interaction	1225.5	1452.0	18.0	-	113.3	-	-	-	15.0	24.0	28.0	-	-	-
	Publishing link	%	8.3%	-	2.9%	16.7%	-	-	-	-	-	-	-	-	-	8.3%
		Interaction	458.0	-	81.0	928.0	-	-	-	-	-	-	-	-	-	32.0
	Text	%	-	-	-	-	-	4.8%	-	100.0%	-	-	-	-	-	-
		Interaction	-	-	-	-	-	38.0	-	0.0	-	-	-	-	-	-
Un	Image	%	81.2%	85.1%	84.1%	73.1%	96.9%	63.4%	41.7%	-	61.5%	100.0%	100.0%	100.0%	75.0%	97.7%

		Interaction	607.1	668.9	23.7	5332.8	149.2	81.9	20.6	-	31.5	11.3	56.2	5.3	1599.3	159.1
	Video	%	2.1%	14.9%	6.1%	11.5%	3.15	4.2%	-	-	15.4%	-	-	-	-	-
		Interaction	92.0	314.6	47.9	12651.0	168.2	63.3	-	-	13.5	-	-	-	-	-
	Publishing link	%	16.7%	-	6.8%	15.4%	-	9.9%	58.3%	-	23.1%	-	-	-	-	2.3%
		Interaction	303.6	-	15.7	6523.5	-	30.6	4.3	-	20.3	-	-	-	-	42.0
	Text	%	-	-	3.0%	-	-	22.5%	-	100.0%	-	-	-	-	25.0%	-
		Interaction	-	-	8.5	-	-	131.7	-	0.0	-	-	-	-	459.0	-
**Encouragement to Take Action**	Image	%	38.6%	65.3%	63.3%	19.2%	77.2%	60.2%	22.2%	-	31.6%	-	72.2%	74.5%	-	91.6%
		Interaction	631.9	897.4	24.7	12177.0	189.9	75.2	190.8	-	97.5	-	49.2	6.2	-	66.1
	Video	%	15.9%	34.7%	15.7%	50.0%	22.8%	12.5%	-	-	36.8%	-	16.7%	24.5%	100.0%	4.2%
		Interaction	342.1	525.8	36.8	6824.3	159.1	149.3	-	-	21.6	-	88.8	4.8	261.0	26.0
	Publishing link	%	45.5%	-	14.7%	30.7%	-	5.7%	77.8%	-	31.6%	-	11.1%	-	-	4.2%
		Interaction	269.4	-	10.0	6746.0	-	31.8	0.9	-	24.2	-	44.5	-	-	46.0
	Text	%	-	-	6.3%	-	-	21.6%	-	100.0%	-	-	-	-	-	-
		Interaction	-	-	19.6	-	-	64.5	-	0.0	-	-	-	-	-	-
**Fear**	Image	%	82.1%	67.4%	74.3%	62.5%	97.2%	59.1%	-	-	66.7%	100.0%	100.0%	100.0%	-	100.0%
		Interaction	256.4	484.9	24.8	6710.8	139.5	48.0	-	-	23.0	16.0	43.7	5.8	-	234.0
	Video	%	-	32.6%	14.3%	25.0%	2.8%	-	-	-	-	-	-	-	-	-
		Interaction	-	449.6	60.0	3556.0	512.0	-	-	-	-	-	-	-	-	-
	Publishing link	%	17.9%	-	11.4%	12.5%	-	9.1%	-	-	-	-	-	-	-	-
		Interaction	316.0	-	11.0	4360.0	-	125.0	-	-	-	-	-	-	-	-
	Text	%	-	-	-	-	-	31.8%	-	100.0%	33.3%	-	-	-	-	-
		Interaction	-	-	-	-	-	59.6	-	0.0	10.0	-	-	-	-	-
**Epidemiological Context**	Image	%	68.5%	83.3%	79.7%	82.2%	100.0%	98.6%	100.0%	-	-	-	-	-	78.0%	100.0%
		Interaction	161.1	600.6	13.5	3594.9	230.0	69.5	0.4	-	-	-	-	-	187.1	14.0
	Video	%	28.2%	16.6%	20.0%	17.5%	-	-	-	-	-	-	-	-	11.3%	-
		Interaction	654.3	550.0	21.7	3131.6	-	-	-	-	-	-	-	-	204.0	-
	Publishing link	%	3.3%	-	0.3%	0.3%	-	0.3%	-	-	-	-	-	-	6.0%	-
		Interaction	108.1	-	4.0	1372.0	-	72.0	-	-	-	-	-	-	106.6	-
	Text	%	-	-	-	-	-	1.0%	-	100.0%	-	-	-	-	4.7%	-
		Interaction	-	-	-	-	-	102.3	-	0.0	-	-	-	-	891.3	-

**Regulatory Measures**	Image	%	15.0%	81.2%	63.9%	100.0%	100.0%	47.8%	21.4%	-	-	-	100.0%	100.0%	15.4%	75.0%
		Interaction	369.7	1737.9	22.6	4322.8	92.4	31.3	2.3	-	-	-	282.0	9.0	31.5	59.1
	Video	%	-	18.8%	1.4%	-	-	-	-	-	-	-	-	-	-	16.7%
		Interaction	-	561.0	1.0	-	-	-	-	-	-	-	-	-	-	7.0
	Publishing link	%	85.0%	-	30.6%	-	-	4.3%	78.6%	-	100.0%	-	-	-	15.4%	8.3%
		Interaction	236.1	-	6.3	-	-	87.0	2.1	-	10.0	-	-	-	109.5	56.0
	Text	%	-	-	4.2%	-	-	47.8%	-	100.0%	-	-	-	-	69.2%	-
		Interaction	-	-	6.7	-	-	44.1	-	0.0	-	-	-	-	365.9	
**Organizational Strategies**	Image	%	22.2%	100.0%	64.9%	-	-	-	-	-	-	-	-	-	50.0%	100.0%
		Interaction	535.5	522.7	11.1	-	-	-	-	-	-	-	-	-	56.5	12.0
	Video	%	-	-	-	-	-	-	-	-	-	-	-	-	-	-
		Interaction	-	-	-	-	-	-	-	-	-	-	-	-	-	-
	Publishing link	%	77.8%	-	27.0%	100.0%	-	20.0%	-	-	-	-	-	-	-	-
		Interaction	120.2	-	6.2	2287.5	-	59.0	-	-	-	-	-	-	-	-
	Text	%	-	-	8.1%	-	-	80.0%	-	-	-	-	-	-	50.0%	-
		Interaction	-	-	6.7	-	-	63.3	-	-	-	-	-	-	87.0	-
**Logistic and Pandemic Management Policies**	Image	%	7.4%	90.9%	58.2%	66.7%	100.0%	18.2%	45.0%	-	42.9%	100.0%	17.6%	100.0%	76.9%	100.0%
		Interaction	395.0	1056.8	17.0	38260.5	82.5	56.5	31.9	-	36.0	38.7	52.0	25.0	638.2	145.8
	Video	%	3.7%	9.1%	-	-	-	9.1%	-	-	-	-	35.3%	-	-	-
		Interaction	120.5	664.3	-	-	-	87.0	-	-	-	-	5474.3	-	-	-
	Publishing link	%	88.9%	-	30.7%	33.3%	-	18.2%	55.0%	-	57.1%	-	47.1%	-	7.7%	-
		Interaction	115.4	-	5.0	5805.0	-	19.0	3.5	-	91.8	-	31.1	-	11.0	-
	Text	%	-	-	11.1%	-	-	54.5%	-	100.0%	-	-	-	-	15.4%	-
		Interaction	-	-	10.9	-	-	44.5	-	0.1	-	-	-	-	272.5	-
**Others**	Image	%	21.1%	87.9%	90.9%	100.0%	96.2%	71.4%	41.7%	-	28.6%	-	20.0%	100.0%	50.0%	75.0%
		Interaction	1521.0	626.3	17.0	3162.4	91.2	42.5	35.6	-	25.0	-	54.5	6.3	248.5	50.8
	Video	%	-	12.1%	1.3%	-	3.8%	7.1%	-	-	-	-	40.0%	-	-	12.5%
		Interaction	-	836.0	11.0	-	80.0	50.0	-	-	-	-	75.0	-	-	22.0
	Publishing link	%	78.9%	-	5.2%	-	-	14.3%	58.3%	-	71.4%	-	40.0%	-	-	12.5%
		Interaction	150.5	-	5.5	-	-	25.0	2.6	-	19.4	-	29.5	-	-	20.0
	Text	%	-	-	2.6%	-	-	7.1%	-	100.0%	-	-	-	-	50.0%	-

		Interaction	-	-	4.5	-	-	40.0	-	0.0	-	-	-	-	564.0	-
**Total of themes**	Image	%	59.6%	76.2%	73.3%	78.2%	90.9%	81.1%	79.3%	-	42.1%	90.9%	69.2%	90.4%	72.0%	97.0%
		Interaction	274.4	710.3	19.9	4185.8	22.9	71.1	6.9	-	45.5	22.9	57.0	5.8	248.1	50.6
	Video	%	18.7%	23.8%	11.3%	17.9%	9.1%	3.0%	-	-	21..0%	9.1%	15.9%	9.6%	9.5%	1.2%
		Interaction	632.6	457.0	31.2	4146.1	24.0	121.7	-	-	19.3	24.0	1982.8	4.8	207.2	15.5
	Publishing link	%	21.7%	-	11.5%	3.9%	-	3.9%	20.7%	-	35.1%	-	14.9%	-	6.4%	1.8%
		Interaction	177.2	-	8.8	5394.6	-	46.5	2.6	-	34.5	-	34.1	-	99.1	47.0
	Text	%	-	-	3.9%	-	-	12.0%	-	100.0%	1.8%	-	-	-	12.2%	-
		Interaction	-	-	13.6	-	-	75.6	-	0.0	10.0	-	-	-	514.7	-

ARS-Alentejo - Regional Health Administration of Alentejo; ARS-Algarve - Regional Health Administration of Algarve; ARS-Lisbon and Tagus Valley - Regional Health Administration of Lisbon and Tagus Valley; DGS - Directorate-General of Health; DRS-Azores - Regional Directorate for Health of the Azores; DRS-Madeira - Regional Directorate for Health of the Autonomous Region of Madeira; SNS - National Health Service.
